# Supplementary figures and images for: The Modular Adaptive Ribosome
Source: PLoS One. 2016 Nov 3;11(11):e0166021. doi: 10.1371/journal.pone.0166021 (PMC5094737; doi:10.1371/journal.pone.0166021)

**Figure S1**

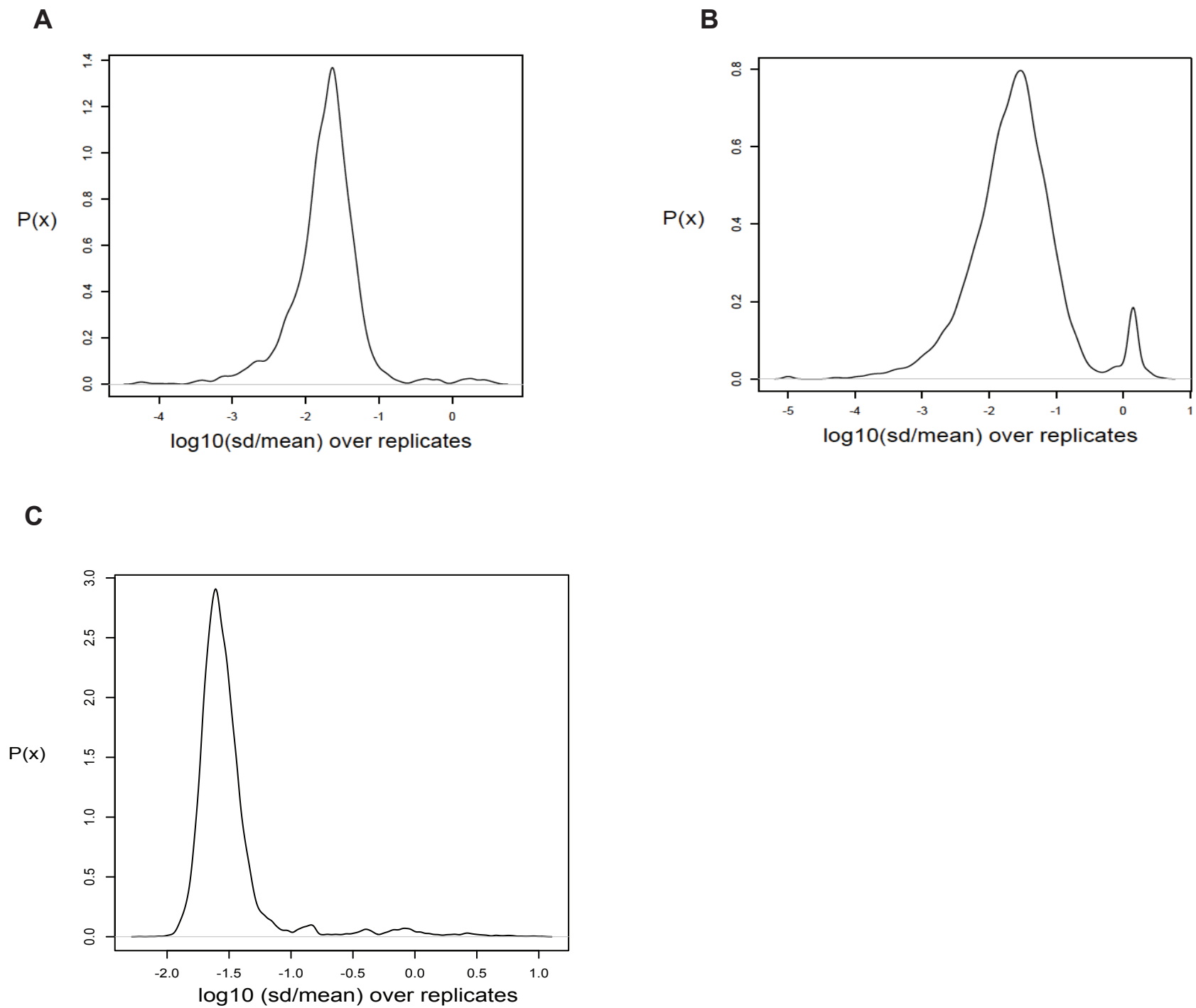

Supplement: S1 Fig — ENCODE data was normalized by median subtraction per array and then log transformed (see Methods). The mean and standard deviation (sd) over replicates was computed to obtain x = log10(sd/mean). The distribution of x for human (A) and mouse (B) ENCODE data and (C) human GTEx data was used to determine the cutoffs xh, xm and xg for reliability of the ENCODE data for human, mouse and GTEx data for humans, respectively. Gene-tissue pairs for which xh > -0.4, xm > -0.6 and xg > 0.1 were excluded from the analysis. (PDF) [file pone.0166021.s001.pdf]

Figure S2

A

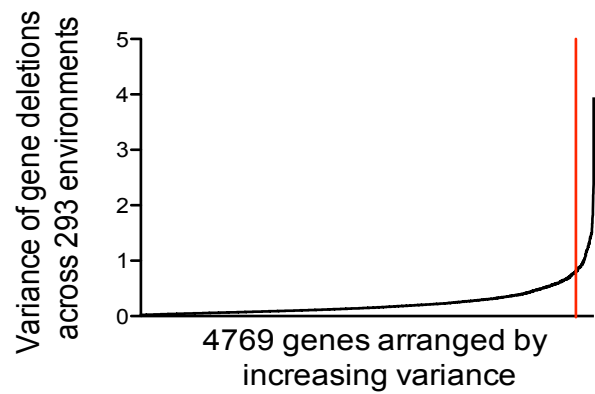

B

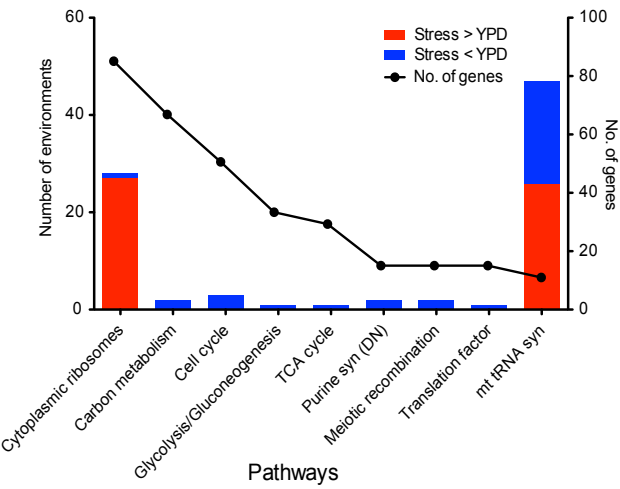

C

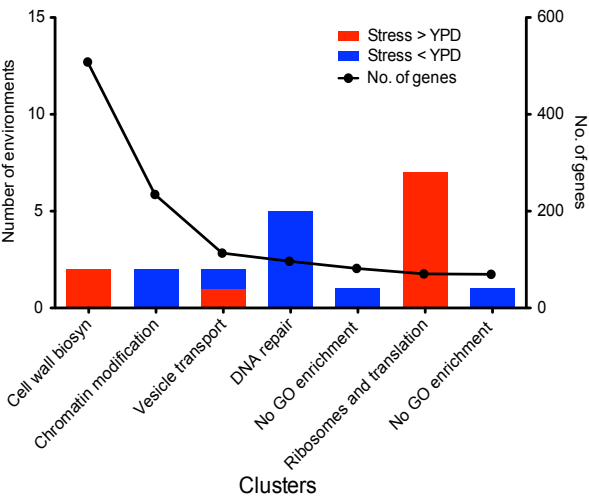

Supplement: S2 Fig — (A) Distribution of variance of normalized growth of all non-essential genes in yeast (4,769) in 293 different environments. The genes are on the x-axis, arranged in increasing order of the variance of normalized growth in 293 environments (y-axis) due to their deletion. The 191 genes to the right of the red line have variance greater than 0.8. (B) Stacked histogram showing the number of cases when the variance of growth from deletion of genes in various pathways was greater or smaller in stress compared to YPD. Gene deletions in the cytoplasmic ribosome and mitochondrial tRNA synthesis pathways had the highest variance in stress compared to YPD. However, in mitochondrial tRNA synthesis pathway genes, the variance is greater or smaller in stress compared to YPD for equal numbers of genes. Only in the ribosomal pathway is the variance in stress conditions greater than YPD for all genes. (C) This figure shows the same data as in B but with the genes stratified into clusters based on their double deletion interactions (see Methods). The ribosomal cluster has the highest variance in stress compared to YPD. (PDF) [file pone.0166021.s002.pdf]

**Figure S3**

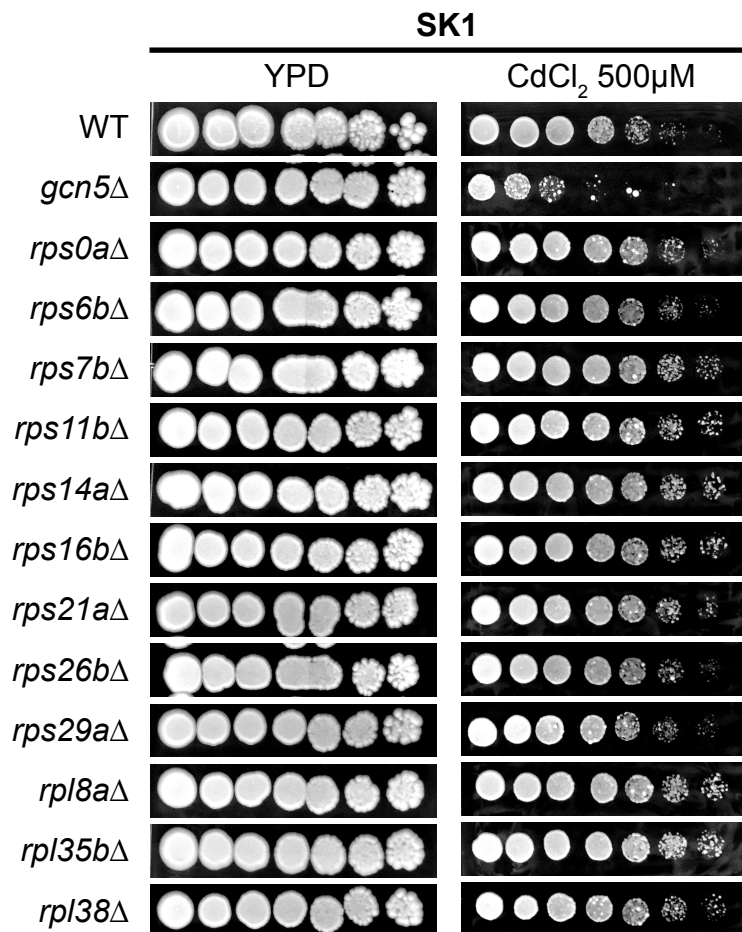

Supplement: S3 Fig — Ten-fold spot dilutions series (starting with 108 cells/ml) of wild type and ribosomal protein deletion strains of SK1 background phenotyped in rich medium YPD and an oxidative stress, Cadmium chloride (CdCl2 500 μM) (PDF) [file pone.0166021.s003.pdf]

Figure S4

**A**

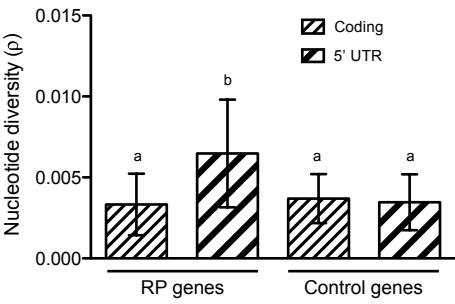

**B**

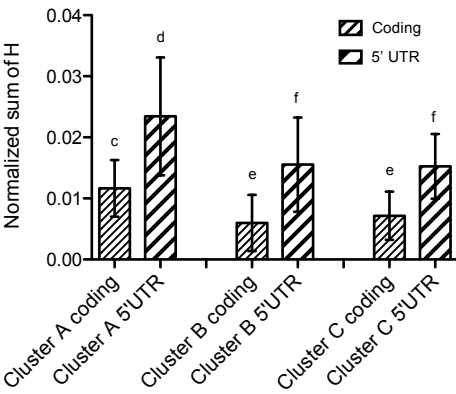

Supplement: S4 Fig — (A) Nucleotide diversity of coding and promoter sequences of ribosomal proteins and a control set of genes using Tukey’s multiple comparisons’ test (P < 0.05). The bars with the same letter code do not differ significantly. (B) Normalized Shannon Entropy of coding region and 5’UTRs of Cluster A, B and C from Fig 2B. Bars with the same letter code do not differ significantly (Tukey’s multiple comparisons’ test, P < 0.05). The figure shows that: (i) The 5’UTR regions of the ribosomal protein sequences are most variable compared to their coding region as well as the 5’UTR and coding regions of the control set of genes; (ii) The 5’UTRs of all the ribosomal proteins in the three clusters are significantly more variable (P < 0.01) than their coding regions; (iii). Proteins in Cluster A have significantly more variable coding regions than clusters B and C (P < 0.05 and P < 0.01 respectively); (iv). Proteins in Cluster A have significantly more variable 5’UTR than Clusters B and C (P < 0.05 and P < 0.01 respectively). (PDF) [file pone.0166021.s004.pdf]

Figure S5

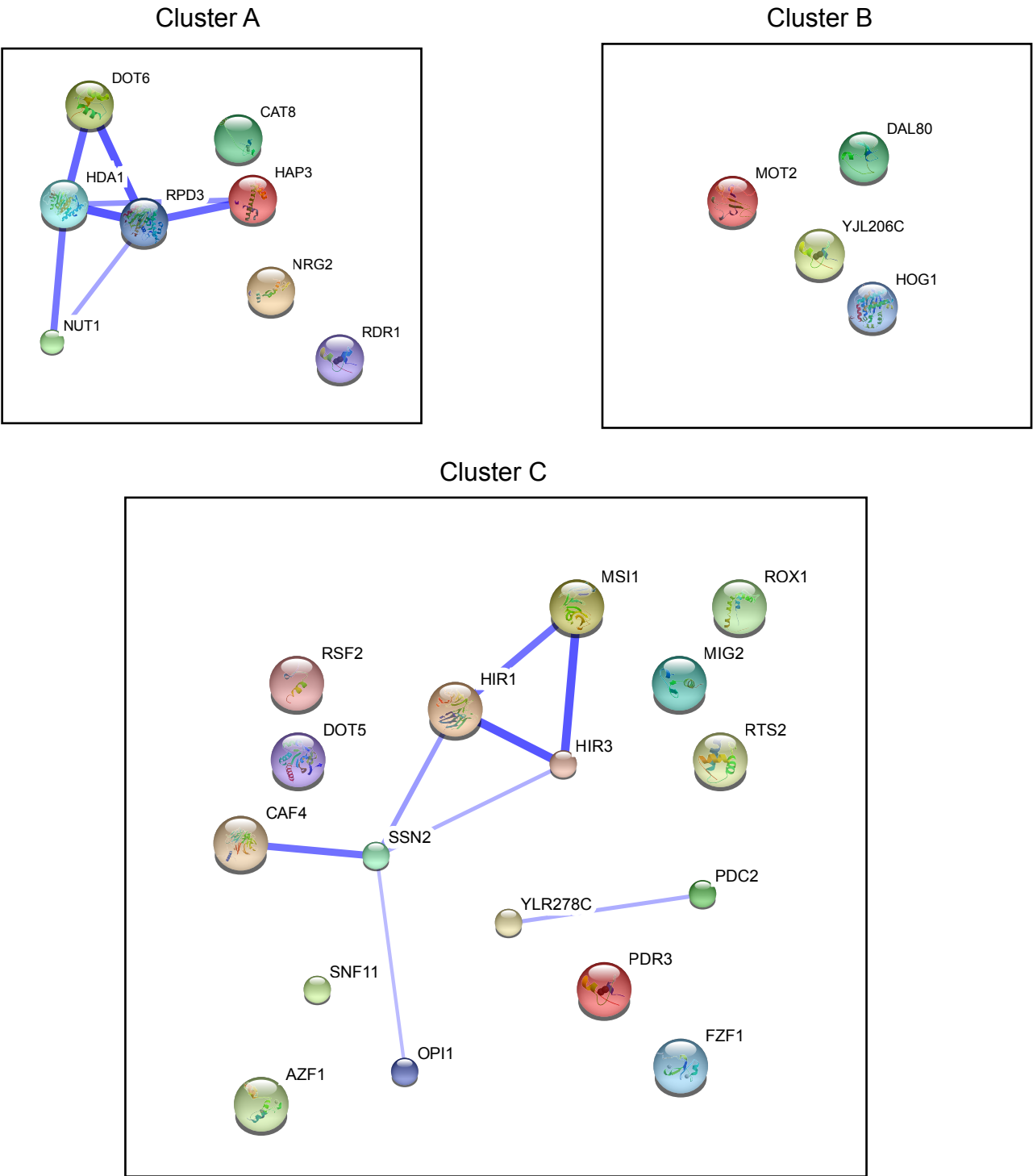

Supplement: S5 Fig — Networks of transcription factors that bind uniquely to ribosomal proteins in Cluster A, B and C (see Fig 2B). These network clusters were identified using the STRING database (http://string-db.org). The thickness of blue lines connecting two transcription factors indicates the strength of experimental evidence for their interaction. Gene enrichment (P < 0.001) of transcription factors in Cluster A is for Histone Deacetylase Complex and in Cluster C for the HIR Complex. (PDF) [file pone.0166021.s005.pdf]

Figure S6

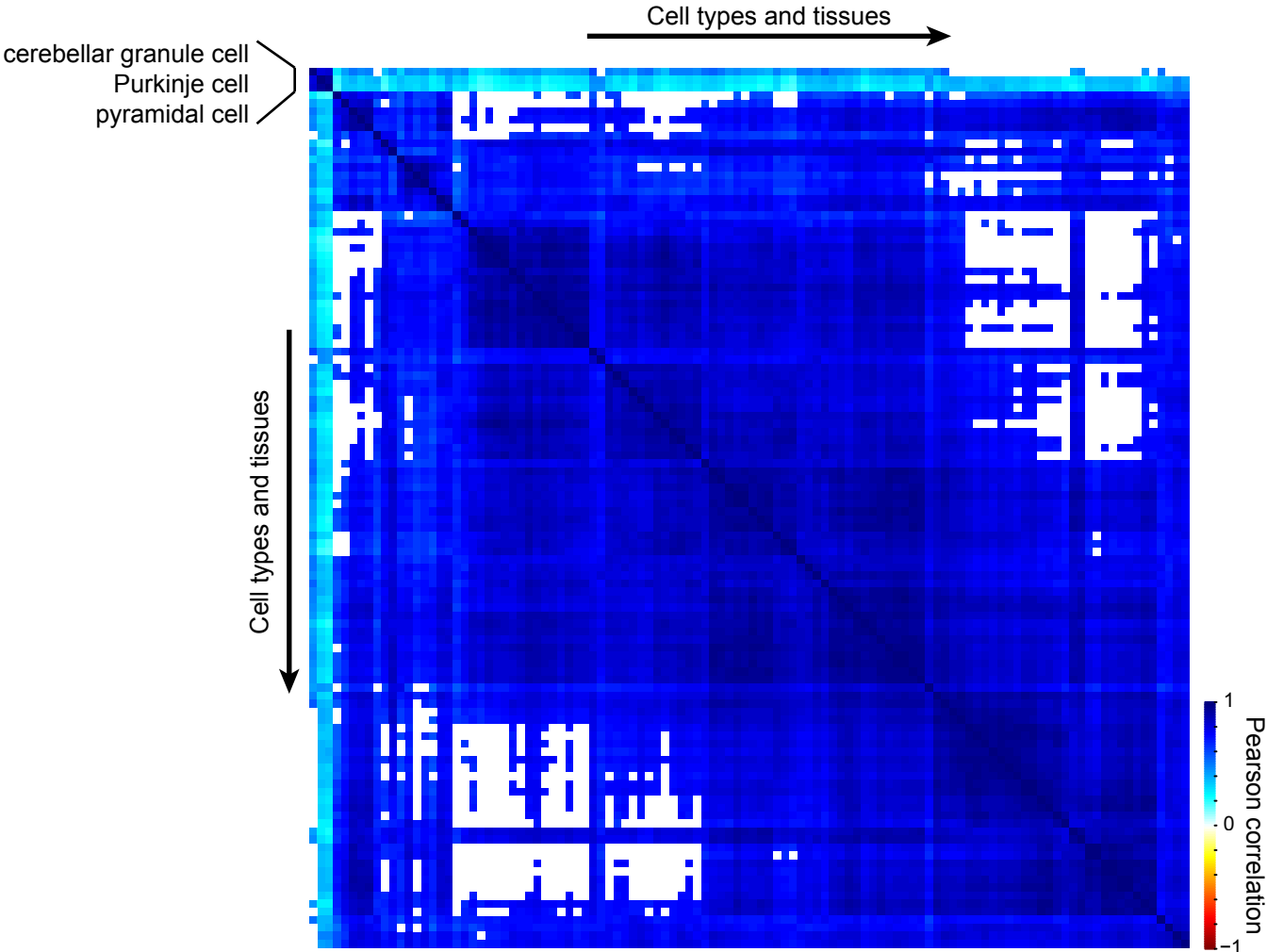

Supplement: S6 Fig — (PDF) [file pone.0166021.s006.pdf]

Figure S7

A

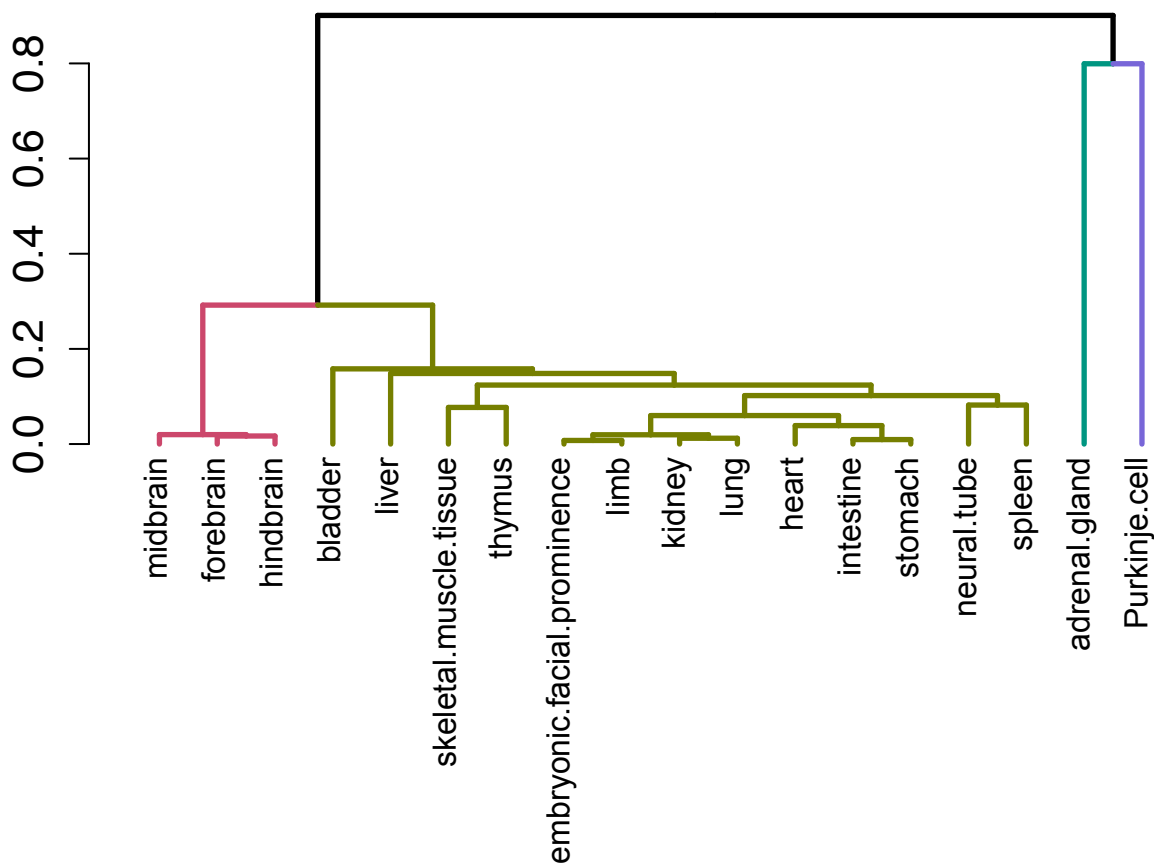

B

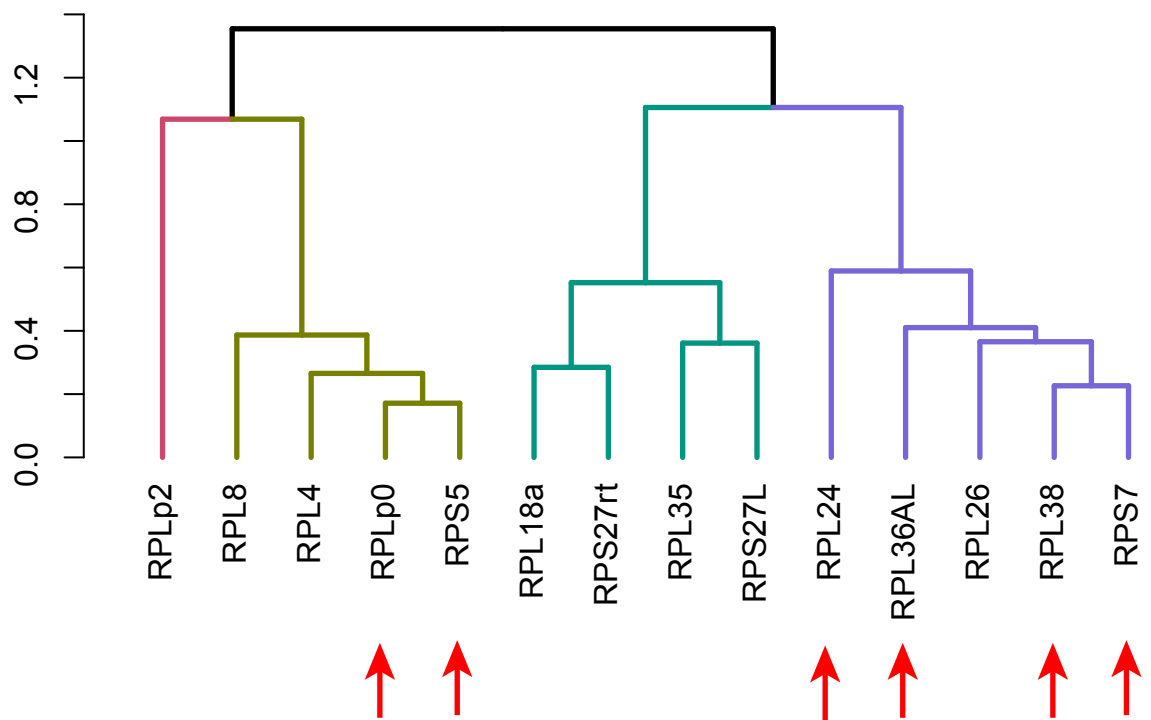

Supplement: S7 Fig — (A) Hierarchical clustering of 18 tissues in mice based on expression rank orders of 14 variable ribosomal proteins (1,000 bootstraps). (B) Hierarchical clustering of the 14 variable ribosomal proteins based on their expression rank orders in 18 tissues in mice (1,000 bootstraps). The ribosomal proteins form three distinct clusters indicated in different colors. The red arrows indicate ribosomal proteins that are also variable in human ENCODE and GTEx data. (PDF) [file pone.0166021.s007.pdf]
